# Application of the mirror technique for block-face scanning electron microscopy

**DOI:** 10.1007/s00429-022-02506-w

**Published:** 2022-05-28

**Authors:** Petra Talapka, Bence Béla Bába, Zoltán Mészár, Réka Eszter Kisvárday, Zsolt Kocsis, Mohit Srivastava, Zoltán Kisvárday

**Affiliations:** 1 Neuroscience Research Group, MTA-Debreceni Egyetem, Debrecen, 4032 Hungary; 2 Department of Anatomy and Embryology, Ruhr-University Bochum, 44801 Bochum, Germany

**Keywords:** Serial block-face EM (SBEM), Double immunostaining, Interneurons, Dendrites

## Abstract

The mirror technique adapted for electron microscopy allows correlating neuronal structures across the cutting plane of adjoining light microscopic sections which, however, have a limited thickness, typically less than 100 µm (Talapka et al. in Front Neuroanat, 2021, 10.3389/fnana.2021.652422). Here, we extend the mirror technique for tissue blocks in the millimeter range and demonstrate compatibility with serial block-face electron microscopy (SBEM). An essential step of the methodological improvement regards the recognition that unbound resin must be removed from the tissue surface to gain visibility of surface structures. To this, the tissue block was placed on absorbent paper during the curing process. In this way, neuronal cell bodies could be unequivocally identified using epi-illumination and confocal microscopy. Thus, the layout of cell bodies which were cut by the sectioning plane can be correlated with the layout of their complementary part in the adjoining section processed for immunohistochemistry. The modified mirror technique obviates the spatial limit in investigating synaptology of neurochemically identified structures such as neuronal processes, dendrites and axons.

**Supplementary Information:**

The online version contains supplementary material available at 10.1007/s00429-022-02506-w.

## Introduction

Each dendrite integrates the activity of a large number of synapses that determines the output of the parent cell. It is known that the computational algorithm of synaptic integration depends on a wealth of spatio-temporal parameters of pre- and postsynaptic structures (Goetz et al. 2021). However, most neuron models rely on assumptions even when basic parameters such as density of excitatory vs. inhibitory synapses should be taken into account due to scarcity of essential structural and functional parameters. As a consequence, point neuron models prevail and are obliged to employ overt simplification for calculating output activity (for review, see Tzilivaki et al. 2019). From a functional point of view, the many synapses acting on the dendrites of a neuron are assumed not only to form an integrative system but also to interact with each other (Hu and Vervaeke 2018; Poirazi and Papoutsi 2020). In this process, precision in terms of timing and voltage parameters is likely to be significant and often goes beyond the ability of neuronal models which use estimations and averaging due largely to lack of precise data. The structural constraints of synaptic connections have been traditionally studied using transmission electron microscopy (TEM). A clear advantage of TEM is that the visualized structures can be paralleled with functional properties such as asymmetric (type I) versus symmetric (type II) synapses being presumed excitatory versus inhibitory role, respectively (Colonier 1968; Gray 1959). TEM is commonly used for disclosing synaptic input–output connectivity characteristics of identified structures, for example, labeled boutons, dendrites and cell bodies. Typically, single ultrathin sections or a short section series is examined. While such an approach could unravel invaluable synaptic information for the population of structures, the precise spatial relationship between pre- and postsynaptic components could not be tackled for the entire length of dendritic processes. From this perspective, distal dendrites have long been in the focus of interest since little is known about their role and contribution in shaping the output activity of neurons. In this regard, synapse type and distribution on the dendritic surface represent important parameters that determine dendritic signal interaction and propagation (Shepherd et al. 1985). Recent advancements in volume electron microscopic applications (FIB-SEM, SBEM, for rev., see Briggmann and Bock 2012) allow examination of large tissue samples as compared with traditional TEM. SBEM is particularly suited to image tissue volume in the range of mm that matches the spatial scale of most dendrites. On the other hand, a major challenge remains how to exploit the benefit of SBEM on neurochemically identified structures. Neurochemical approaches commonly compromise membrane integrity which, however, is an essential requirement for electron microscopy (EM) including SBEM. Recent attempts tried to solve the above issue. Using the fluorescent signal of the labeled structures, the sections were subjected to confocal- and light microscopy (LM), after fixation, followed by collecting serial EM images with the help of fiducial landmarks (Maclachlan et al. 2018). Another study used the mirror technique that employed adjoining sections with cut cell bodies on the mirror surfaces. In one section, immunohistochemistry was carried out to visualize the neurochemical marker and in the other section, the complement of the cell bodies and the emerging dendrites were reconstructed using serial section TEM (Talapka et al. 2021). While both reports managed to preserve high-quality ultrastructure, the alignment process required the use of light microscopic sections which have a limited thickness, typically, not more than 100 µm. The spatial constraint of the above methods does not favor the full reconstruction of dendrites which often run several hundreds of µm or longer. The method presented here is an adaption of the mirror technique (Talapka et al. 2021) which offers 3D-electron microscopic reconstruction of structures in a large tissue volume, for example, the synaptome of entire dendrites. It is based on the identification of neuronal cell bodies in the surface of thick tissue blocks using confocal reflectance imaging instead of LM of sections. This method opens new vistas to carry out combined light- and electron microscopic analysis on neurochemically identified structures for providing quantitative measures of synapse organization of specific neuron types.

## Materials and methods

Animals were maintained and bred under appropriately controlled conditions with the approval of the local ethics committee for animal research studies at the University of Debrecen in line with European Union guidelines for the care of laboratory animals (Directive 2010/63/EU).

### Tissue fixation and sectioning

Anesthetized (Urethane, 1.5 g/kg) 14-week-old C57BL/6 J male mice (*n* = 6) were transcardially perfused with Tyrode’s solution (gassed O_2_; pH = 7.2) for 1 min. For DAB-based immunolabelling, the fixative contained 2% paraformaldehyde, 1% glutaraldehyde and 15% (v/v) saturated picric acid in 0.1 M phosphate buffer (PB; pH 7.4) for 40 min (rate: 3 ml/min). Then, the brain was removed and stored in the same fixative at 4 °C overnight. For double-fluorescent immunolabelling, the fixative contained 2% paraformaldehyde and 0.5% glutaraldehyde in 0.1 M phosphate buffer (PB; pH = 7.4) for 40 min (rate: 3 ml/min) followed by 0.1 M PB wash (10 min). Then, the brain was removed and stored in PB at room temperature (RT; 22–23 °C).

After rinsing the brain 3 times in 0.1 M PB (pH = 7.4), large tissue blocks (4 × 4 × 4 mm^3^) containing the primary visual cortex area (VISp), see Allen Mouse Brain Atlas; Lein et al. 2007) were dissected using a Mouse Brain Matrice (Electron Microscopy Sciences, Mouse Coronal, 69,090). The bottom of the block representing the white matter was glued (superglue) on the metal chuck of the vibratome (Leica, VT 1000S). Consequently, the sectioning plane was parallel to the cortical surface. Then, the block was submerged in the buffer container of the vibratome filled with 0.1 M PB at RT.

### Preparing blocks and sections

**Fig. 1:**
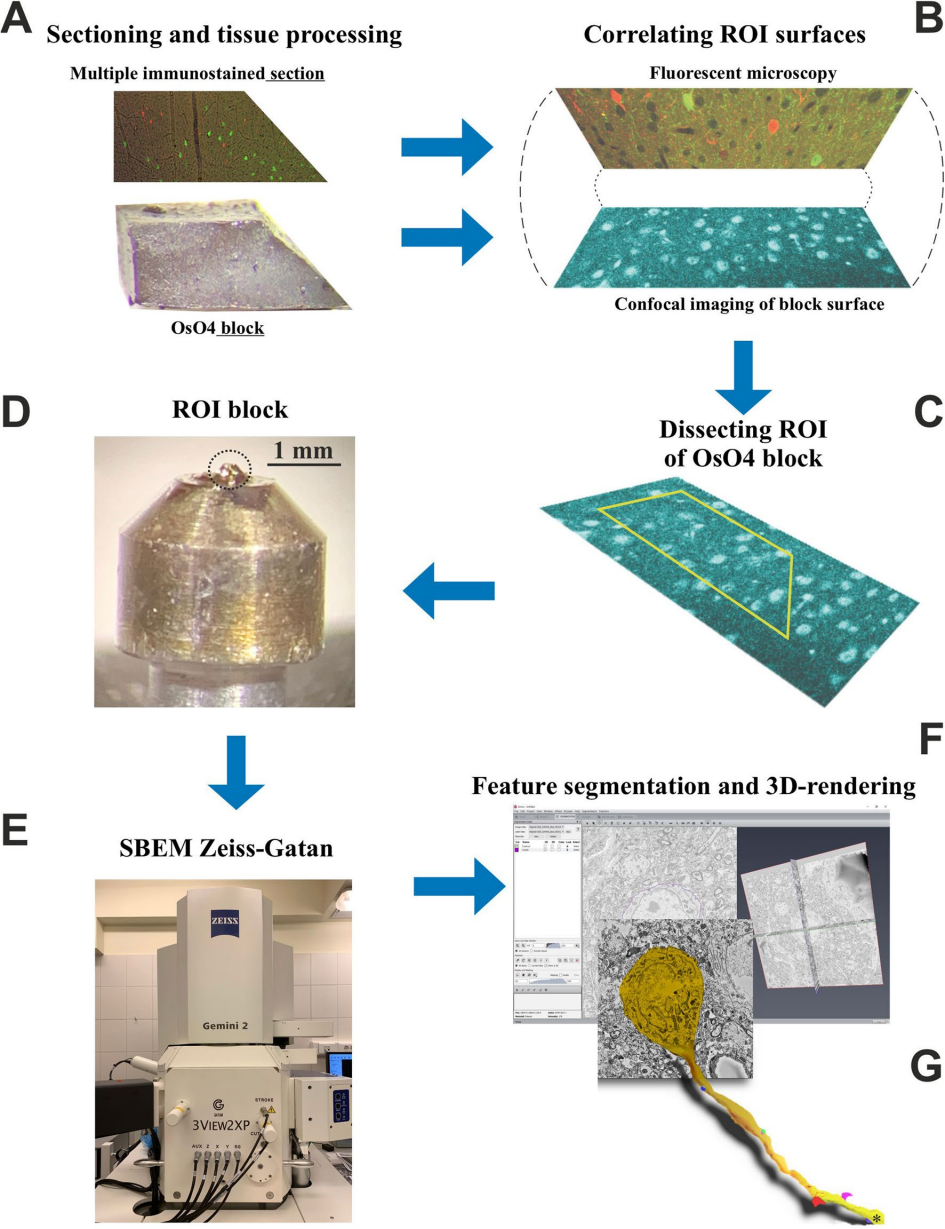
Workflow of the mirror technique adapted for SBEM showing the main steps. **A** Adjoining or mirror surfaces of a section and a tissue block containing neuronal cell bodies which are cut in two parts by the sectioning plane. Immunohistochemistry can be applied to the section for revealing neuron specific markers in those cell bodies while the block can be prepared for examining the dendritic processes of the same cell bodies using SBEM without compromising the quality of ultrastructure. **B** Confocal reflectance image reveals surface structures whereby cell bodies and fiducial landmarks can be correlated (identified) with structures in the immunostained section. **C** After identification of immunopositive cell bodies in the OsO_4_ block, the ROI is defined. **D** Mounting of the block containing the ROI on an aluminum pin. **E** Serial section block-face scanning electron microscopy is performed for tracing processes of neurochemically identified cell bodies. **F** Segmentation of pre- and postsynaptic structures across SEM images and rendering them in 3D **G**

For increasing tissue sample size, from the large block, several smaller blocks and adjoining sections were obtained in the following way. Several parallel slits were made perpendicular to the block surface using a sharp tip surgical blade (BB511, Braun, Germany, S.Fig. 1). The final shape of the resulting tissue slabs was trapezoid to be able to identify their sides. The first sectioning was carried out 400–600 µm below the block surface corresponding to the boundary between layer 3 and 4 in VISp. Next, a single 80–100 µm section was collected, after that, section thickness was increased to pick up a 600–800-µm-thick block. The latter block contained the granular and infragranular layers. Thereby, the above sectioning approach produced two blocks flanking an 80–100-µm-thick section with two mirror surfaces, one facing towards the upper block and one towards the lower. Then, the blocks were processed for SEM while the section underwent immunohistochemistry (see below).

### Single-immunostaining using DAB as chromogen

Polyclonal somatostatin (SOM) antibody was used for labeling cortical interneurons (INs). All steps were carried out at RT unless otherwise stated. To reduce endogenous peroxidase activity sections were treated with 1% H_2_O_2_ diluted in 0.05 M Tris-buffered-saline (TBS; pH = 7.6) for 10 min. Then, a mixture of 0.25% bovine-serum-albumin, 0.1 M DL-Lysine (Sigma-Aldrich, L6001) and 10% normal goat serum (Sigma-Aldrich, G9023) in 0.05 M TBS (pH = 7.6) containing 0.05% Triton X-100 (Sigma-Aldrich, X100) was applied (30 min) for suppressing non-specific immunoreactivity. The primary antibody (Rabbit anti-Somatostatin-14, Peninsula Laboratories, T-4103) was used in 1:1000 dilution containing 0.05% Triton X-100 for 1 day during continuous agitation of the sections. The secondary antiserum (biotinylated goat anti-rabbit IgG, VECTOR Laboratories, BA-1000) was used in 1:200 dilution for 4 h, followed by avidin–biotin complexed to horseradish peroxidase (ABC; VECTOR Laboratories, PK-6100) in 1:400 dilution for overnight. Between each step, the sections were washed in 0.05 M TBS (pH = 7.6) for 3 × 10 min. For visualization of the immuno-labeling, they were incubated in 0.05 M 3,3-diaminobenzidine-tetrahydrochloride (DAB; Sigma-Aldrich, D-5637) in 0.05 M Tris Buffer (TB; pH = 7.6) for 10 min. The enzymatic reaction was visualized in the presence of 0.02% H_2_O_2_ for 1–2 min followed by a 2 × 5 min rinse in TB.

### Double-fluorescent immunostaining

Parvalbumin (PV) and calretinin (CR) antibodies were chosen for labeling cortical INs. Pre-embedding immunohistochemistry was performed on 80–100-µm-thick vibratome sections. All steps were carried out at RT unless otherwise stated. To reduce endogenous peroxidase activity, sections were treated with 3% H_2_O_2_ diluted in 0.05 M Tris-buffered-saline (TBS; pH = 7.6) for 20 min. Next, a mixture of 0.25% bovine-serum-albumin, 0.2 M DL-Lysine and 10% normal goat serum (Sigma-Aldrich, G9023) in 0.05 M TBS (pH = 7.6) containing 0.05% Triton X-100 was used (30 min) for suppressing non-specific immunoreactivity. Thereafter, the neuron-type markers, polyclonal antibodies to CR (rabbit, Swant, CR 7697) and PV (guinea pig, Synaptic Systems, 195,004) supplemented with 0.05% Triton X-100 were applied in 1:2000 dilution for 1 day during continuous agitation of the sections. Secondary antisera, goat anti-rabbit IgG conjugated with Alexa Fluor 594 (Invitrogen, A-11037) and goat anti-guinea pig IgG conjugated with Alexa Fluor 488 (Invitrogen, A-11073) and were used in 1:500 dilution for 4 h. Between each step, sections were washed in 0.05 M TBS (pH = 7.6) 3 × 10 min. Mounting of the sections on slides and coverslipping was performed with BrightMount/Plus aqueous media for fluorescent imaging (Abcam, ab103748).

### Postfixation of tissue blocks

A modified heavy metal staining (mHMS) protocol was applied on the 600–800-µm-thick tissue blocks which were subjected for osmium treatment only (Tapia et al. 2012; Kubota et al. 2018) to enhance tissue contrast for SBEM examination. Blocks were washed in 0.15 M cacodylate buffer supplemented with 2 mM calcium-chloride (pH = 7.4) (hereinafter referred to as 0.15 M cacodylate buffer) five times for 5 min. Then, they were post-fixed for 1 h on ice in a solution containing 3% potassium ferrocyanide (dissolved in 0.3 M cacodylate buffer supplemented with 4 mM calcium-chloride) and 2% osmium tetroxide (OsO_4_; dissolved in DW), followed by a rinse in 0.15 M cacodylate buffer (5 × 3 min) and in DW (5 × 3 min). Freshly made 1% thiocarbohydrazide solution (dissolved in DW) was applied at 60 °C for 20 min to bridge the following OsO_4_ impregnation. After a repeated rinse in DW (5 × 3 min), blocks were incubated at RT for 30 min in 2% osmium tetroxide. Following a thorough wash (3 × 5 min) in DW, sections were placed in 1% uranyl acetate solution (dissolved in DW) and incubated overnight at 4 °C. On the next day, Walton’s en bloc lead aspartate staining was performed. Lead aspartate solution was prepared by dissolving 0.066 g of lead nitrate in 10 ml of 0.03 M aspartic acid, pH adjusted to 5.5 with 1 M potassium hydroxide, and kept in an oven at 60 °C for 30 min until dissolved. Sections were washed in DW (3 × 5 min) and placed into the lead aspartate solution at 60 °C for 30 min. Tissue blocks were again rinsed in DW (5 × 3 min), dehydrated in increasing concentration of ethanol (20, 50, 70, 96, 100%, 5 min each) and acetone (2 × 10 min), and embedded in resin overnight followed by curing at 56 °C for 24 h (DurcupanTM ACM; Sigma-Aldrich, 44,610).

### Confocal imaging of the block surface

Confocal imaging confirmed that surface structures and landmarks which are necessary for correlating mirror surfaces can be visualized only in the resin-free part of the block surface. Before imaging, the specimen (SEM block) was placed between a clean glass slide and a coverslip without a mounting medium. For fixating the specimen, a touch of superglue was applied at the four corners of the coverslip to bind to the glass slide for holding the block in between them. Care was taken so that the glue did not contaminate the specimen. In this way, the block could be positioned under the microscope’s objective while keeping its surface naked.

**Fig. 2:**
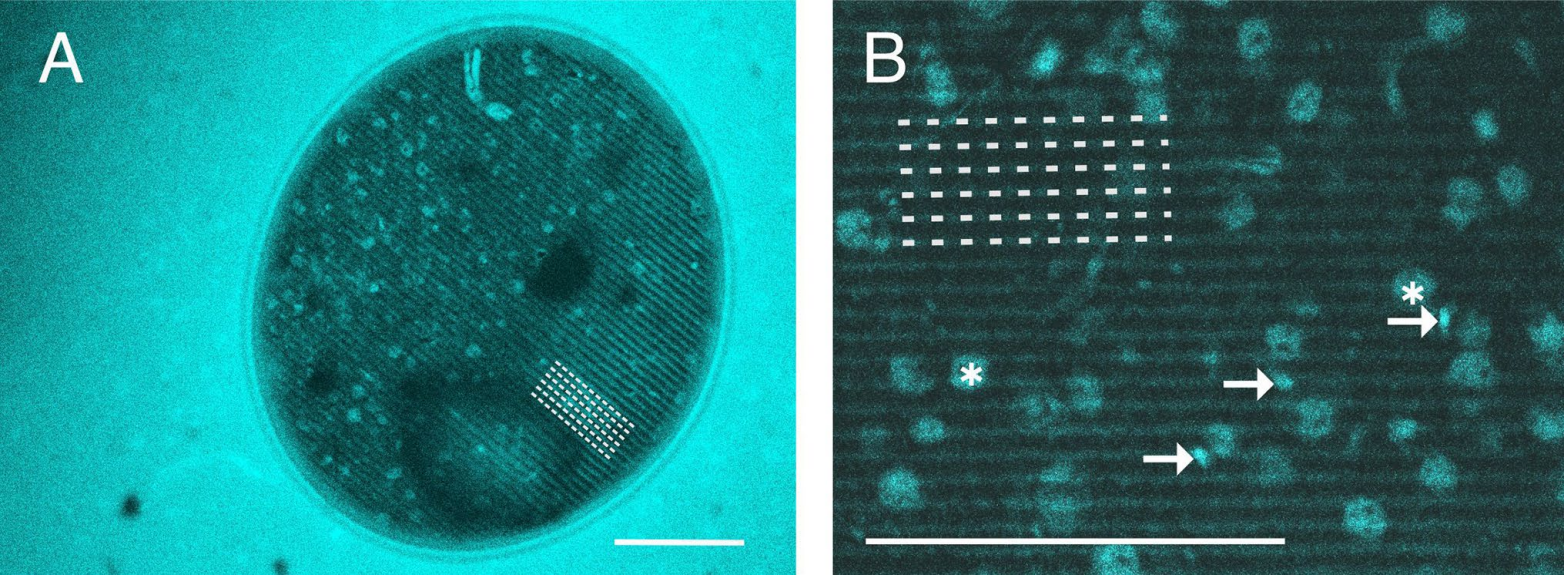
**A** Confocal image of a resin-embedded thick section processed for transmission electron microscopy (see Talapka et al. 2021) using epi-fluorescent detection (excitation/emission at 405/461 nm). The image was taken through a coverslip under which an air-bubble inadvertently became trapped (circular area in the center). Visibility of the section surface through the air bubble is superior compared with the surrounding region. The air covered central part shows the silhouette of cell bodies and blood vessels in the surface, i.e., in the sectioning plane. Note that the stripy pattern represents vibratome chatter marks (broken lines). **B** Verification of the discovery shown in **A** using a 400 µm tissue block prepared without a coverslip (see “Results” and Fig. 3). The type of surface structures can be anticipated by size and shape such as cell bodies (star) and small blood vessels (arrow). Image resolution in **A**: 0.315 µm/pixel, **B**: 0.155 µm/pixel. Scale bars **A**, **B**: 100 µm

Confocal microscopy offers a broad range of imaging modes of which, for scanning the surface of the specimen, the blue laser (405 nm) was chosen for autofluorescent excitation in line scanning mode. The wavelength of the autofluorescent emission ranged between 495 and 95 nm. For epi-imaging, high-quality scans from the upper 1–2 µm of the tissue block could be obtained (Figs. 2B, 4A, 5A, 6A) at 0.155–0.350 µm/pixel resolution. Image scans were captured with a galvanometer confocal scanner (FluoView3000, Olympus) attached to an inverted microscope (IX81, Olympus) equipped with a 10 × objective (UPLSAPO, NA:0.4, Olympus). The images provided ample details for recognizing cell bodies as well as blood vessels. It should be kept in mind, while osmicated sections can be flattened during the dehydration process, tissue blocks having a spatial dimension in the millimeter range lose their planar nature along the cut surfaces due largely to tissue inhomogeneity. For example, fiber- and cell body density differences between cortical layers and white matter vs. gray matter result in uneven surfaces. Hence, several surface images of the same ROI were acquired by changing the focal depth in 2–5 µm steps. Using an image convolution algorithm, the composite surface image was generated that represented all parts of the block surface in a single image plane ((FluoView3000, Olympus, Figs. 4A, 5A, Suppl.Movie).

## Results

### Visualization of the tissue surface in blocks after OsO_4_ postfixation

The recently introduced mirror technique preserves utmost ultrastructure not only in sections but tissue blocks (Fig. 1). For 3D-EM investigation, blocks in the mm range would be favored because they would allow full reconstruction of entire neurons. However, osmium-treated blocks are opaque; hence, transmission LM cannot help in correlating their surface with the mirror surface of the immunostained section. Here, we report a serendipity discovery that led to the adaption of the mirror technique for SBEM. After intense osmium treatment, 80 µm thick sections were embedded in resin, mounted on glass slides and coverslipped (see “Materials and methods”). As expected, the sections were so dark that even high power illumination (100 W halogen bulb) was not sufficient to illuminate through and visualize structures on the surface. Next, high-resolution confocal microscopy was employed using reflectance imaging. The results were disappointing because only faint patches could be recognized on the surface scans, while, no structures in the inner part could be seen at all. However, in one of the sections, an air bubble was trapped accidentally between the section surface and the coverslip (Fig. 2A). Clearly, under the air bubble, the silhouette of distinct structures including cell bodies, blood vessels and tissue debris could be readily recognized (Fig. 2). In addition to tissue components, surface landmarks such as vibratome chatter were also visible indicating that the observed region represented the very surface of the section, i.e., the sectioning plane (broken lines). The obvious conclusion was drawn: visibility of surface structures requires a resin-free condition on the specimen’s surface.

**Fig. 3:**
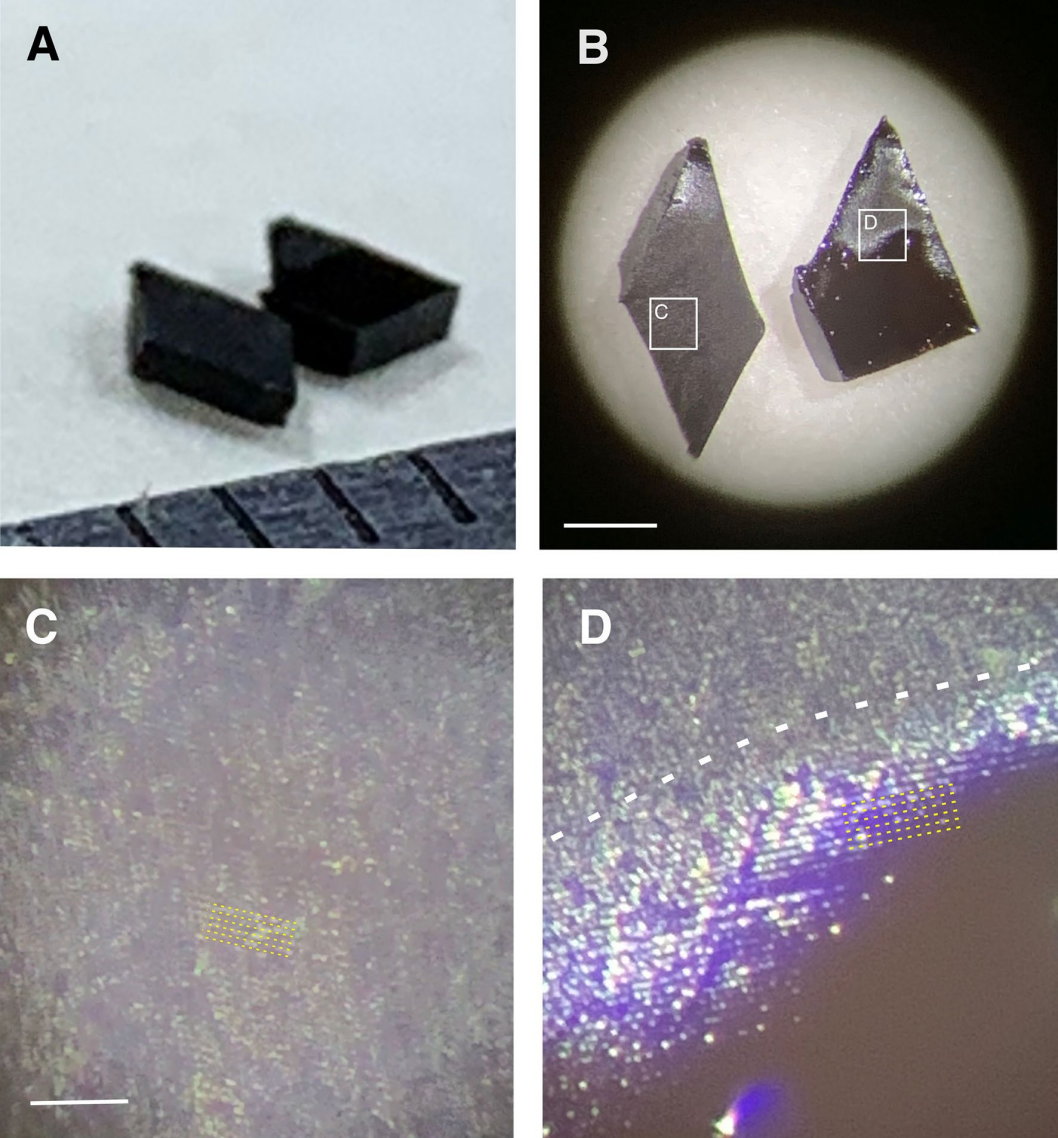
Quality check of tissue blocks before confocal imaging. Block surface must be free of resin otherwise light reflectance prevents visualization of structures. **A** Photograph of two tissue blocks placed on a filter paper after the curing process. Their mirror surface of the blocks is facing upwards. **B** The same two blocks are viewed at higher magnification under a stereo microscope. The left block shows a matt character almost for the entire surface area indicating no excess of resin. On the contrary, the right block shows a shiny character in the lower two-third of the surface indicating remnants of resin causing light reflectance. The framed areas are enlarged in **C** and **D**, respectively. **C** The block surface is free of resin and suitable for revealing surface structures such as contours of cell bodies and fiducial landmarks (see confocal images in Figs. 4, 5, 6, 7). Thin broken lines indicate cutting chatter. **D** The boundary between matt (upper) and shiny (lower) surface zones is denoted by thick broken line. Cutting chatter (thin broken lines) is visible both parts but tissue structures are obscure where resin forms a thin sheet. Scale bars, **A**, **B**: 1 mm, **C**, **D**: 100 µm

To replicate the above condition and prepare osmicated blocks without coverslipping, the following modifications were made in the resin-embedding procedure. After the overnight soaking of the blocks in resin at room temperature, they were transferred on an absorbing filter paper and placed on a hot plate (40 °C). While keeping warm, the excess of resin was removed by gently touching the surface with filter paper strips. After this step, the block was placed on a new sheath of filter paper in a way that the surface to be examined (ROI) was oriented vertically (Suppl.Fig. 2). In this way, draining of the resin was enhanced by gravity. Then, the blocks were transferred in the same orientation in the oven for curing the resin at 56 °C for 24 h. In the ready-made blocks, the resin penetrated all inner parts but the surface was free of resin due to the absorbent effect of the filter paper (Fig. 3A). Such a surface looked non-shiny (Fig. 3B, block in the left side, C). If, however, an excess of the resin remained on the block surface (Fig. 3B, block in the right side, D) the light-reflecting resin-sheet hindered optical access to the underlying structures. Note, that at this stage the specimen is fragile so they have to be handled with care. Furthermore, a dust-free condition is imperative at all steps since contamination cannot be removed readily from the surface. Finally, the ready-made sections or tissue blocks were gently transferred in a storage vial or a beam-capsule.

**Fig. 4:**
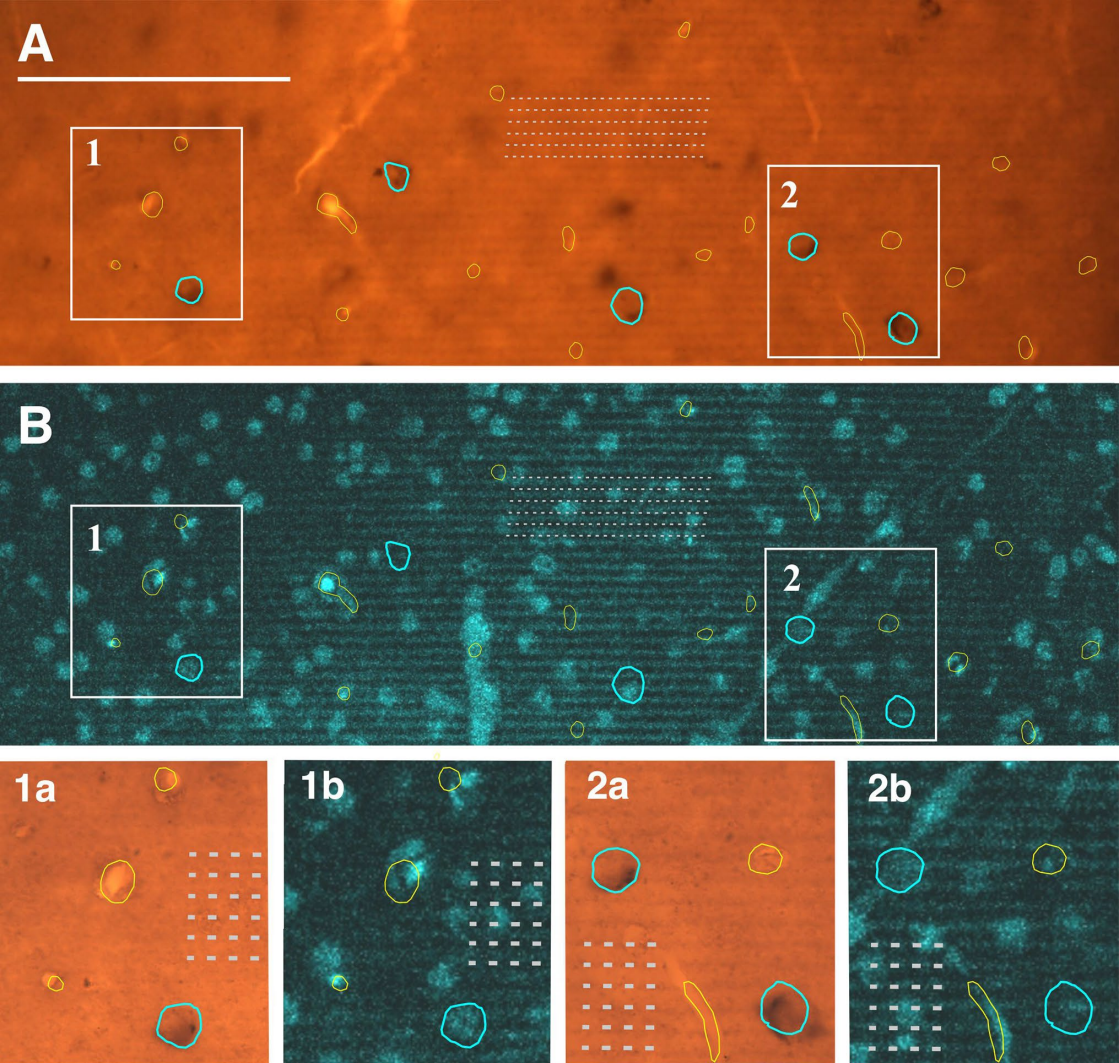
Correlating the mirror surface of the single-immuno-stained (DAB chromogen) section **A** with that of the SEM block using confocal microscopy **B**. Lower panels show the framed areas (1 and 2) pairwise for the immunostaining (1a, 2a) and the SEM block (1b, 2b) at high magnification. Blue contours mark SOM-immunopositive somata, yellow contours denote surface blood vessels, and broken lines indicate vibratome chatter. It should be noted that the slight mismatch between yellow contours indicating blood vessels is due to the difference in tissue treatment (immunohistochemistry vs. osmification alone) which causes some distortion of the matching surfaces. Scale bars: 100 µm

**Fig. 5:**
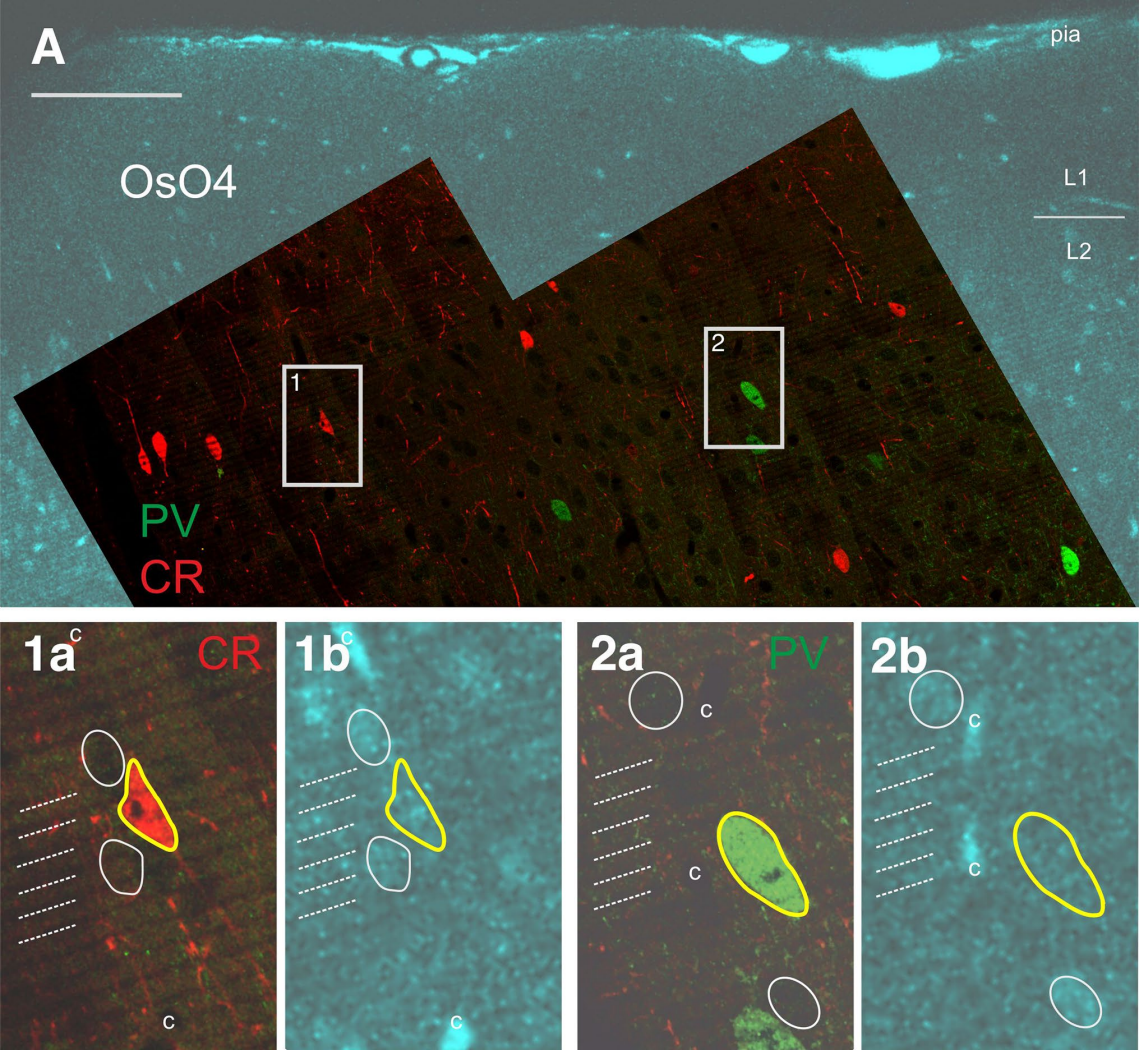
Correlating the mirror surface of the double-immunofluorescent-stained section and the adjoining SEM block. **A** Superimposition of a confocal image taken from the osmium-treated block (turquoise) with the surface image of the double-fluorescent immunostained section (PV: green, CR: red). Lower panels show high power pictures of the framed areas (1 and 2), separately for the immunolabelling (**1a**, **2a**) and the corresponding part of the block surface (**1b**, **2b**). Immunolabelled somata are indicated with yellow contour. For fostering correlation between the mirror surfaces, the silhouette of non-immunostained cell bodies (white contour), blood vessels (c) and the pattern of vibratome chatter (broken lines) are also marked. Scale bar: 100 µm

**Fig. 6:**
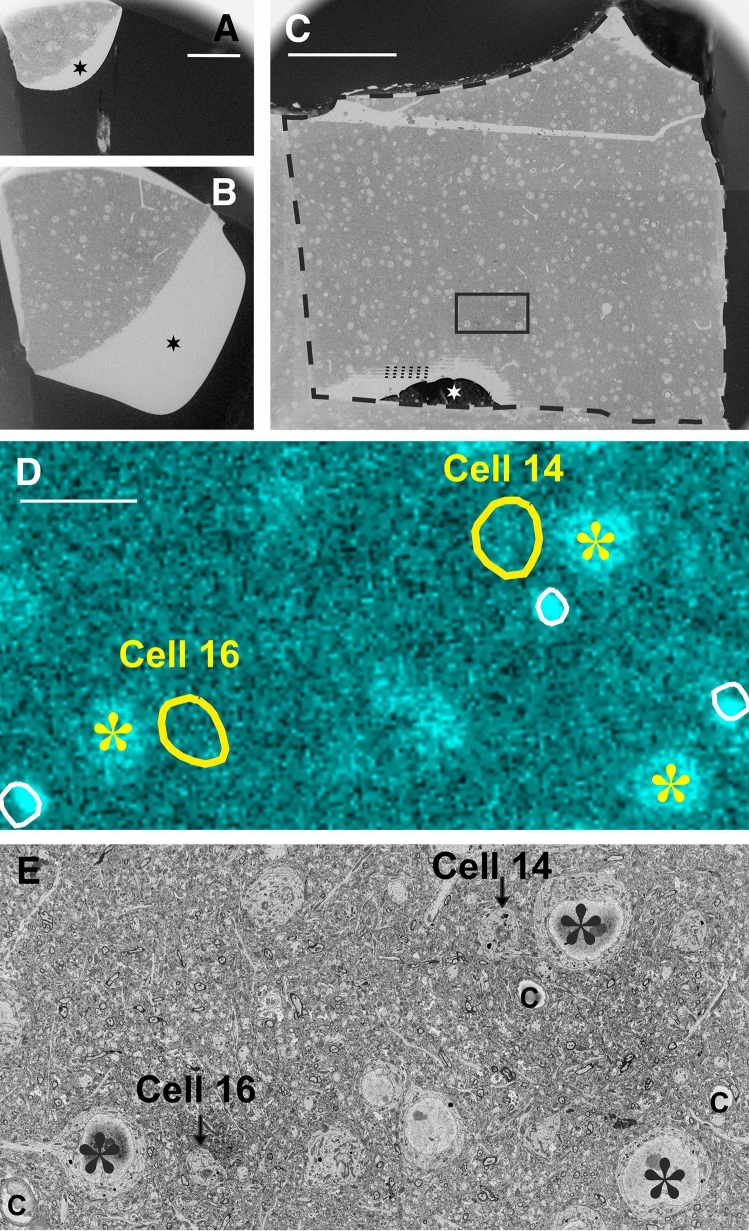
Determining the location of neurochemically identified somata in the SEM. **A** and **B** Surface images of the same block at different z-level. Black surrounding represents gold sputter, blank zone (star) corresponds to inadvertent resin spill during mounting of the block on the aluminum pin. **C** SEM image of the same block. The framed area indicates ROI containing two SOM-immunopositive cell bodies (see below). Block edge is indicated by thick broken line, star marks the remnant of gold sputter and thin broken lines denote the pattern of vibratome chatter. The empty zone between gold sputter and vibratome chatter represents resin spill. The ROI was taken approximately 2 µm below the block surface. **D** Confocal image of the ROI comprising two SOM-immunopositive somata (yellow contours of Cell #14 and #16) and fiducial landmarks such as capillaries (white contour) and nearby immunonegative cell bodies (asterisks). **E** The same ROI with two SOM-immunopositive somata (arrows) and landmarks such as capillaries (c) and immunonegative cell bodies (asterisks) are seen under the SEM. Scale bars: **A**, **B**: 10 µm, **C**: 50 µm, **D**, **E**: 20 µm

### Matching mirror surfaces

The next step was to match the block surface with the corresponding, also named as the mirror surface of the immunostained section. For this purpose, the contour of immuno-labeled neurons and nearby fiducial landmarks such as blood vessels was drawn in the sectioning plane (see Talapka et al. 2021). To disambiguate contours of closely neighboring somata, including those of labeled and non-labeled ones, care was taken to draw all surface contours with high precision using 100 ×  oil objective. Figures 4 and 5 provide, respectively, examples for matching single- and double-immunostained sections with the mirror surface of SEM blocks. For single-immunostaining, DAB was used as a chromogen, whereas for double-immunostaining, green and red fluorescence tagged antibodies. Using DAB, the contours of labeled somata as well as fiducial landmarks could be recognized unequivocally (Fig. 4). Using fluorescent probes, cell body labeling was also unambiguous and surface contours, including cutting chatter, could be readily recognized (Fig. 5). However, immuno-negative somata and blood vessels were less discernible under fluorescence illumination, and each shows, typically, a dark patch.

After drawing the contour of surface structures in the immunostained section, the reconstruction is overlaid with the mirror image of the SEM block. This step is shown in Fig. 4 for two ROIs in a SOM-immunostained section (panels 1a, 2a) and corresponding regions of the SEM block (panels 1b, 2b). The constellation of the selected contours designates a perfect match between the mirror surfaces and, consequently, determines the location of immunopositive cell bodies and their dendrites to be examined in the SEM block. The same strategy was applied for the double-immunofluorescent specimen shown in Fig. 5. The two ROIs (#1, #2) containing a CR- and a PV-labeled neuronal cell body, respectively, were selected in layer 3. High magnification images of the immunolabelling (panels 1a, 2a) and those of the mirror surfaces (panels 1b, 2b) reveal a perfect match between the tissue components including fiducial landmarks. Admittedly, no double-labeled neurons were found in this part of the immunostained section (Fig. 5A). A reasonable explanation could be that the proportion of double-labeled cells is quite low in V1 (ref.) and that only those neurons can be considered which are located on the surface.

### Mounting of the block

Once the ROI was determined and the neuronal cell bodies were identified in the mirror surfaces, the block was glued onto an aluminum pin (Gatan Inc., Pleasanton, CA, USA) using resin. For a better quality of SEM images, the electrical conductivity of the specimen was increased by sputter coating of gold (5 nm thickness). Trimming the block was carried out when the size exceeded the spatial recommendation of the ultracut device (3View2XP, Gatan, Pleasanton, CA, USA). In such a case, trimming was performed before sputter coating (Q300T, Quorum Technologies, UK).

### SBEM imaging

**Fig. 7:**
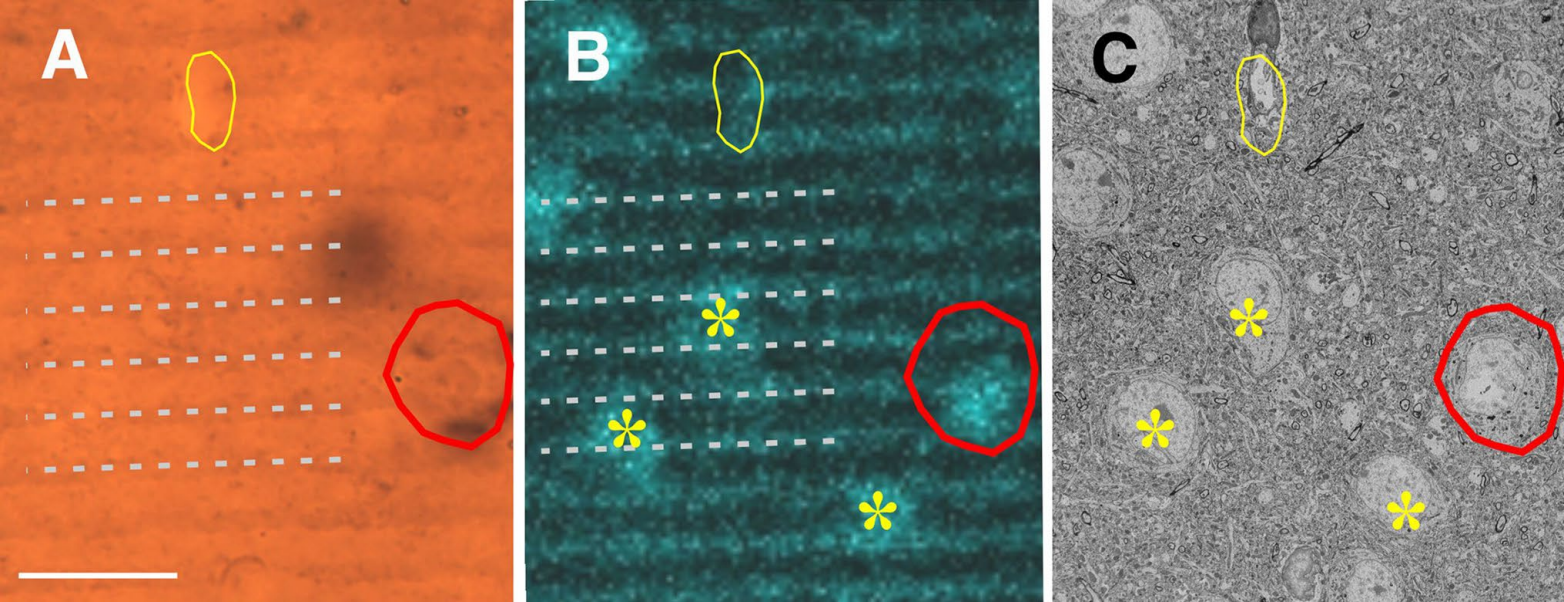
Identifying immunolabelled cell bodies in the SEM using the mirror technique. Correlating light microscopy **A**, confocal imaging **B** and SEM **C** is based on the layout of the visualized structures as exemplified for a SOM-immunopositive cell body (red contour), non-immunolabelled somata (asterisks), a blood capillary (yellow contour) and surface chatter (broken lines). Central in the process that confocal microscopy enables a detailed surface view of heavily osmicated tissue blocks, but only if their surface is free of resin (see “Materials and methods”). Scale bar (**A**–**C**): 20 µm

The next step was to find the ROI in the SEM image. To this end, sectioning of the block was made step-by-step. As approaching the surface, section thickness was set 100–200 nm initially to speed up the process. It should be noted here that the block surface is typically non-planar; therefore, the gold sputter was reached first at the highest part of the block which does not necessarily represent the ROI. Hence, after each stroke of the knife, a quick scan (low resolution) of the entire block surface was made to follow the progress in exposing the ROI. Having reached the location of ROI, section thickness was set to 50 nm. Representative images of the approaching phase are shown in Fig. 6A, B When the preselected somata, i.e., the complement of immunopositive cell bodies, could be identified in the ROI, the collection of high-resolution SEM images commenced. Such a case is illustrated in Fig. 6C, D for two SOM-immunopositive cell bodies. Their position concerning fiducial landmarks and other neighboring somata (non-immunostained) could be identified unequivocally (Fig. 7).

### Tracing of dendrites

**Fig. 8:**
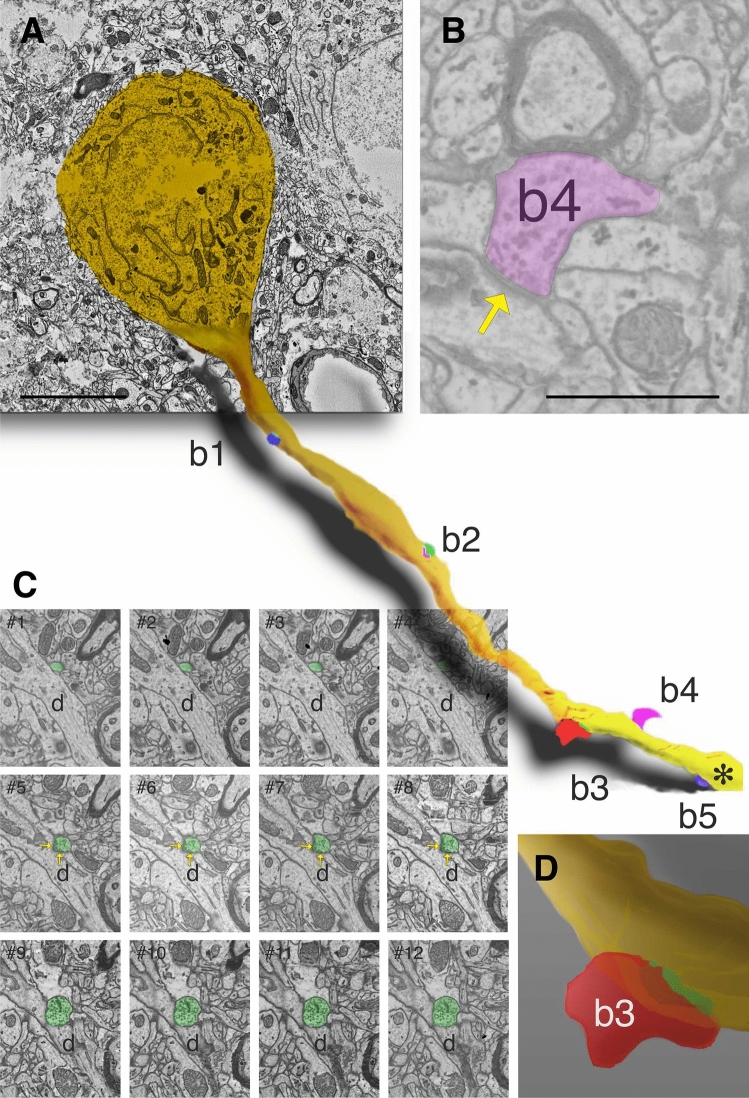
3D-reconstruction of SEM images in layer 3 of VISp. **A** Cell body of a somatostatin immunopositive neuron and one of the emerging dendrites which receives synapses from 5 boutons (b1–b5). The reconstruction represents only the proximal part of the dendrite whose truncated part is indicated by an asterisk. **B** One of the boutons b4 established a type II synapse (arrow) on the dendrite. **C** Series of SEM images (#1–12) showing b3 (green) which established a synapse (arrow in #5–8) with the dendritic shaft (d). **D** An enlarged view of the 3D rendering of b3 showing the symmetric contact (green) with the same dendrite: **A**, 5 µm, **B**, 1 µm.

The selected cell body was scanned at low resolution (8 × 8 nm) for identifying the emergence of a dendritic shaft. For speeding up the searching process, only every fifth level was taken for scanning until the first dendritic shaft appeared. Then, the dendritic shaft and its surrounding ambience were scanned at each z-level using high image resolution (4 × 4 nm per image pixel). For increasing the efficacy of image acquisition, the ROI size was set large enough to trace the dendritic profile without repositioning across 10–20 consecutive levels, each level representing 50 nm section thickness. The benefit of these considerations could shorten imaging time and reduce data volume. Finally, for tracing more than one dendrite, an array of ROIs was organized using multiple ROIs whose scanning parameters (size, resolution, and pixel time) were individually set (Digital Micrograph, GMS software 3.2, Gatan Inc, USA). The resulting images were organized in stacks, scaled and reconstructed in 3D using the Amira program package (ThermoFisher, Scientific Inc. MA, USA) as illustrated in Fig. 8. For 3D rendering of the reconstruction, an open source creation software suite was employed (Blender 2.92.).

## Discussion

Recent works aiming to unveil synaptic interrelations between neurons have gained impetus from technical advancements regarding image acquisition approaches as well as 3D-reconstruction systems. In this regard, serial section EM has become the gold standard for reconstructing ultrastructural features in a large volume of biological tissues including the nervous system (Denk and Horstmann. 2004; Knott et al. 2009; Micheva and Smith 2007; Bock et al. 2011; Helmstaedter 2013). While structural details are invaluable for unraveling connectivity aspects of neuron networks their complexity cannot be understood without taking into account the chemical nature of the components (Fishell and Kepecs 2020). Traditionally, immunohistochemistry is the method of choice. However, immunohistochemistry imposes a compromise on tissue ultrastructure quality whereby small diameter structures cannot be traced and reconstructed for long distances since any discontinuity in the plasma membrane may hinder unequivocal identification. Therefore, membrane integrity is of utmost importance for large-scale electron microscopic reconstructions.

Here, we introduce a solution to the above problem using a modified version of the mirror technique (Talapka et al. 2021) that allows tracing neurochemically characterized structures in tissue blocks instead of sections. The modified technique can directly address questions regarding the distribution of synapses along entire dendrites with little or no limit in dendritic length. Followed from this, quantitative measures and topographic relationships of synapses on entire dendrites can be obtained which, in turn, are key parameters for understanding how synapses are organized along the dendritic surface and determine the function of dendrites (Kirchner and Grigorjeva 2021).

### Previous attempts

In a recent report, Talapka et al. (2021) introduced an application of the mirror technique using 60–80-µm-thick adjoining immunostained and osmicated sections, respectively. The rationale of the technique is based on the idea that the specimen should be split into two parts (sections), one part is used for LM labeling and the other part only for EM, thereby the quality of ultrastructure does not have to suffer from those reagents and solutions which are necessary for immunohistochemistry. To this end, neurons whose cell body is cut by the sectioning plane are processed independently. Importantly, the mirror technique allows direct proof in terms of the neurochemical identity of the structures and their synaptology through the cell body.

Similar considerations led (Maclachlan et al. 2018) to carry out SEM reconstruction of fluorescent axons without the need for histochemical conversion of the labeling. Generally, for EM, the fluorescent signal is converted into DAB deposit which, however, requires a histochemical protocol that is detrimental for ultrastructure. Therefore, they used confocal imaging of fiducial landmarks (blood vessels and section edges) for determining the location of genetically expressed fluorescent signals in boutons before preparing the same section for SEM. It should be noted that the above studies aimed to preserve high fidelity ultrastructure, although the technical solution they reported was quite different. While the former study used partitioning of the specimen (two adjoining sections) and separated the histochemical treatment from the SEM protocol, the latter study did thorough documentation of the structures in the confocal microscope before applying the SEM protocol on the same section.

### Light- and electron microscopic correlation

Targeted electron microscopic investigation of neurochemically identified dendrites was developed recently using the mirror technique (Talapka et al. 2021). According to this approach, the osmicated section must be transparent for enabling matching the location of the cell body which was cut by the sectioning plane. The mirror technique thus limits the size of the EM block according to the thickness of the osmicated section, and hence, the feasibility to trace several hundred micrometer (µm) long dendrites. On the other hand, the modified mirror technique described here can cope with blocks not only LM sections for processing for EM. The modified mirror technique rests on the recognition that the surface of osmium-treated tissue can be imaged using confocal microscopy although the tissue block is opaque. Provided that the excess resin is removed thoroughly from the surface of the specimen, confocal imaging can capture structural details such as the contour of cell bodies and blood vessels. Importantly, the thickness of the tissue does not play a role in obtaining those structural details. This is an advantage compared with that of using sections of limited thickness allowing not only a precise correlation between the mirror surfaces but to conduct volume EM of tissue blocks even in the size of millimeters. Importantly, such a large-scale serial section ultrastructural investigation is now applicable for studying entire dendrites with known neurochemical content.

### Spatial considerations

In the cerebral cortex, neurons possess polarized morphology—shape, branching pattern of the processes—all of which have been traditionally used for classification. In this regard, most dendrites run quasi-perpendicular to the brain surface which is quite evident for the apical dendrite of pyramidal cells (Kanari et al. 2019) but also for non-pyramidal cells (Jin et al. 2001). From the perspective of tissue blocks used for the modified mirror technique, it is important to consider the orientation of the sectioning plane for the following reasons. First, synapses are best visualized when the pre- and postsynaptic site and the synaptic cleft are viewed in a plane perpendicular to that of the plasma membrane. Translated for dendrites, the most advantageous sectioning plane is axial which, considering that most dendrites prefer a radial orientation to the cortical plane, corresponds to a plane that is parallel to the cortical surface (see Talapka et al. 2021).

An inherent limitation of the modified mirror technique is that only those dendrites of a given immunopositive neuronal cell body (cut on the surface) can be analyzed in 3D-SEM which are present in the adjoining osmium-treated block (see Suppl.Fig. 1). In turn, dendrites which emerge from the cell body-half present in the immunostained section are unlikely to be traced without discontinuity due to the fact of its compromised ultrastructure (continuous tracing of thin dendrites requires utmost quality regarding ultrastructure). No such limitation applies for the blocks where a superb ultrastructure can be achieved. In addition to this, an important question regarding the spatial constraints of the method is what proportion of the dendrites of the selected neuron can be analyzed. The answer depends on the particular dendritic field of the neuron type and the block size. For example, using the horizontal sectioning plane across the cell body of a pyramidal cell the upper tissue block (towards the cortical surface) will contain largely the apical dendrites whereas the lower tissue block chiefly the basal dendrites but not both. A similar consideration can be made for the inhibitory neuron types. For example, a bipolar cell (Prönneke et al. 2015; Sohn et al. 2016) that has typically an equal number (length) of dendrites above and below the cell body the horizontal sectioning plane is likely to give rise to a 50% representation of all dendrites either in the upper or in the lower block. Contrary to this, there are interneurons of the bitufted and Martinotti types which possess more (longer) dendrites above the cell body and hence more dendrites will be present in the upper block than in the lower block.

Another important question is how the method can cope with the lateral span of dendrites. The answer depends on two parameters, the spatial extent of dendrites from the parent soma on the one hand, and the xy dimension of the tissue block used for 3D-SEM on the other hand. In this regard, the dendritic field of most neurons in the cortex can be confined to a cylinder of 200–400 µm in diameter. Since the size of the tissue block to be imaged in the SEM can be up to 2000 × 1200 µm in the xy plane (maximum size for sectioning area using the Gatan ultramicrotome device), imaging and reconstruction of the dendrites is not limited by their lateral spread. Hence, the modified mirror technique introduced here allows the reconstruction of entire dendrites.

Finally, using immunostained sections for identifying the neurochemical character only one of the blocks, either the upper or the lower one can be used for the very same cell. This is because section thickness, typically 60–80 µm, far exceeds the diameter of neuronal cell bodies. If, however, section thickness was in the range of a few µm no such a restriction applies. Pertinently, vibratome sectioning is not suited to achieve sections which are thin enough, however, genetically modified specimen does not necessitates of using immunostaining at all and thus both adjoining blocks can be used comprising all dendrites of the same cell (see below).

### Timing aspects

The actual time cost of any method is one of the important factors for applicability. In this regard, the modified mirror technique does not require extra time compared with its predecessor that is based on sections instead of tissue blocks. It was noticed that confocal imaging which necessary for matching immunostained section with the EM block is relatively fast step because it is supported by custom friendly softwares of the confocal microscope system, for example, for merging image scans of the block surface (see Suppl.Movie). The most time-consuming part remains the SEM imaging and post-processing including segmentation of the structures and 3D-data analysis (Titze et al. 2018).

### Present and future potentials

The modified mirror technique extends the investigation repertoire of neurochemically identified structures at the ultrastructural level from medium to large-scale 3D analysis. With the continuous advancement of using genetically expressed neurochemical identification, it is anticipated that the mirror technique can be further improved and employed for specimens without spatial restriction. Recent transgenic studies have achieved selective visualization of neuron types opening new vistas in exploiting the use of smart engineering of the genetic content. For example, the fluorescent protein, tdTomato can be transgenically expressed in the GABAergic neuron population (Besser et al. 2015; He et al. 2016) revealing inhibitory neurons. Such a manipulation in the genetic content offers simplification of the mirror technique by omitting the immunohistochemical step and correlate cell body position along both sides of the sectioning plane according to the fluorescent signal. Furthermore, the experimental paradigm would not only become simpler but the two blocks representing the mirror surfaces permit full-scale reconstruction of all dendrites of the very same neuron (see Suppl.Fig. 1C without the immunostained section). Similar arguments will apply for axons without the necessity to subjecting to immunohistochemical labeling. Last but not least, recent development in genetic approaches allows disentangling subtypes of a neuronal cell population using solely intersectional transcriptomics. In this way, co-expression of multiple features can be visualized based on fluorescent reporters using genetic and viral labeling approaches and, as mentioned above, without the need for immunohistochemistry (see He et al. 2016; Tasic et al. 2016; Zeisel et al. 2015; Lein et al. 2007).

## Summary and conclusions

The mirror technique for tissue blocks described here provides a solution to the spatial limitation of sections. Consequently, neuronal processes such as dendrites and axons can be traced and reconstructed in 3D in a much larger tissue volume than a section can offer. There are three technical points to be noted which represent newly added features with the potential to study the presynaptic repertoire of neurochemically identified dendrites. First, tissue blocks instead of sections can be investigated with SBEM instead of TEM which allows semi-automatic image acquisition for large tissue volume. Second, SBEM images do not suffer from sectioning artifacts such as non-linear distortions providing a fault-free data acquisition. Third, the modified mirror technique is compatible with multiple labeling for specific markers including immunohistochemical as well as a transgenic expression for molecular profiling (Rees et al. 2017).

## Supplementary Information

Below is the link to the electronic supplementary material.

Supplementary file1 (JPG 304 kb)

Supplementary file2 (JPG 284 kb)

Supplementary file3 (MP4 4164 kb)

## Data Availability

The data that support the findings of this study are available from the corresponding author upon reasonable request.

## References

[CR1] Besser S, Sicker M, Marx G, Winkler U, Eulenburg V (2015). A transgenic mouse line expressing the red fluorescent protein tdTomato in GABaergic neurons. PLoS ONE.

[CR2] Bock DD, Lee WC, Kerlin AM, Andermann ML, Hood G, Wetzel AW (2011). Network anatomy and in vivo physiology of visual cortical neurons. Nature.

[CR3] Briggman KL, Bock DD (2012). Volume electron microscopy for neuronal circuit reconstruction. Curr Opin Neurobiol.

[CR4] Colonnier M (1968). Synaptic pattern on different cell types in the different laminae of the cat visual cortex. Electron Microsc Stud Brain Res.

[CR5] Denk W, Horstmann H (2004). Serial block-face scanning electron microscopy to reconstruct three-dimensional tissue nanostructure. PLoS Biol.

[CR6] Fishell G, Kepecs A (2020). Interneuron types as attractors and controllers. Annu Rev Neurosci.

[CR7] Goetz L, Roth A, Häusser M (2021). Active dendrites enable strong but sparse inputs to determine orientation selectivity. PNAS.

[CR8] Gray EG (1959). Axo-somatic and axo-dendritic synapses of the cerebral cortex: an electron microscope study. J Anat (lond).

[CR9] He M, Tucciarone J, Lee S, Nigro MJ, Kim Y, Levine JM, Kelly SM, Krugikov I, Wu P, Chen Y (2016). Strategies and tools for combinatorial targeting of GABAergic neurons in mouse cerebral cortex. Neuron.

[CR10] Helmstaedter M (2013). Cellular-resolution connectomics: challenges of dense neural circuit reconstruction. Nat Methods.

[CR11] Hu H, Vervaeke K (2018). Synaptic integration in cortical inhibitory neuron dendrites. Neurosci.

[CR12] Jin X, Mathers PH, Szabo G, Katarova Z, Agmon A (2001). vertical bias in dendritic trees of non-pyramidal neocortical neurons expressing GAD67–GFP in vitro. Cereb Cortex.

[CR13] Kanari L, Ramaswamy S, Shi Y, Morand S, Meystre J, Perin R, Abdellah M, Wang Y, Hess K, Markram H (2019). Objective morphological classification of neocortical pyramidal cells. Cereb Cortex.

[CR14] Kirchner JH, Gjorgjieva J (2021). Emergence of local and global synaptic organization on cortical dendrites. Nat Commun.

[CR15] Knott GW, Holtmaat A, Trachtenberg JT, Svoboda K, Welker E (2009). A protocol for preparing GFP-labeled neurons previously imaged in vivo and in slice preparations for light and electron microscopic analysis. Nat Protoc.

[CR16] Kubota Y, Sohn J, Hatada S, Schurr M, Straehle J, Gour A, Neujahr R, Miki T, Mikula S, Kawaguchi Y (2018). A carbon nanotube tape for serial-section electron microscopy of brain ultrastructure. Nat Commun.

[CR17] Lein ES, Hawrylycz MJ, Ao N, Ayres M, Besinger A, Bernard A (2007). Genome-wide atlas of gene expression in the adult mouse brain. Nature.

[CR18] Maclachlan C, Sahlender DA, Hayashi S, Molnár Z, Knott G (2018). Block face scanning electron microscopy of fluorescently labeled axons without using near infra-red branding. Front Neuroanat.

[CR19] Micheva KD, Smith SJ (2007). Array tomography: a new tool for imaging the molecular architecture and ultrastructure of neural circuits. Neuron.

[CR20] Poirazi P, Papoutsi A (2020). Illuminating dendritic function with computational models. Nat Rev Neurosci.

[CR21] Prönneke A, Scheuer B, Wagener RJ, Möck M, Witte M, Staiger JF (2015). Characterizing VIP neurons in the barrel cortex of VIPcre/tdTomato mice reveals layer-specific differences. Cereb Cortex.

[CR22] Rees CL, White CM, Ascoli GA (2017). Neurochemical markers in the mammalian brain: structure, roles in synaptic communication, and pharmacological relevance. Current Med Chem.

[CR23] Shepherd GM, Brayton RK, Segev MJP, I, Rinzel j, Rall W, (1985). Signal enhancement in distal cortical dendrites by means of interactions between active dendritic spines. PNAS.

[CR24] Sohn J, Okamoto S, Kataoka N, Kaneko T, Nakamura K, Hioki H (2016). Differential inputs to the perisomatic and distal-dendritic compartments of VIP-positive neurons in layer 2/3 of the mouse barrel cortex. Front Neuroanat.

[CR25] Talapka P, Zs K, Marsi LD, Szarvas VE, Kisvárday ZF (2021). Application of the mirror technique for three-dimensional electron microscopy of neurochemically identified GABA-ergic dendrites. Front Neuroanat.

[CR26] Tapia JC, Kasthuri N, Hayworth K, Schalek R, Lichtman JW, Smith SJ, Buchanan J (2012). High-contrast en bloc staining of neuronal tissue for field emission scanning electron microscopy. Nat Protoc.

[CR27] Tasic B, Menon V, Nguyen T (2016). Adult mouse cortical cell taxonomy revealed by single cell transcriptomics. Nat Neurosci.

[CR28] Titze B, Genoud C, Friedrich RW (2018). SBEMimage: versatile acquisition control software for serial block-face electron microscopy. Front Neural Circuits.

[CR29] Tzilivaki A, Kastellakis G, Poirazi P (2019). Challenging the point neuron dogma: fs basket cells as 2-stage nonlinear integrators. Nat Commun.

[CR30] Zeisel A, Muňoz-Manchado AB, Codeluppi S (2015). Cell types in the mouse cortex and hippocampus revealed by single-cell RNA-seq. Science.

