# Supplementary figures and images for: Application of the mirror technique for block-face scanning electron microscopy
Source: Brain Struct Funct. 2022 May 28;227(6):1933–47. doi: 10.1007/s00429-022-02506-w (PMC9232443; doi:10.1007/s00429-022-02506-w)

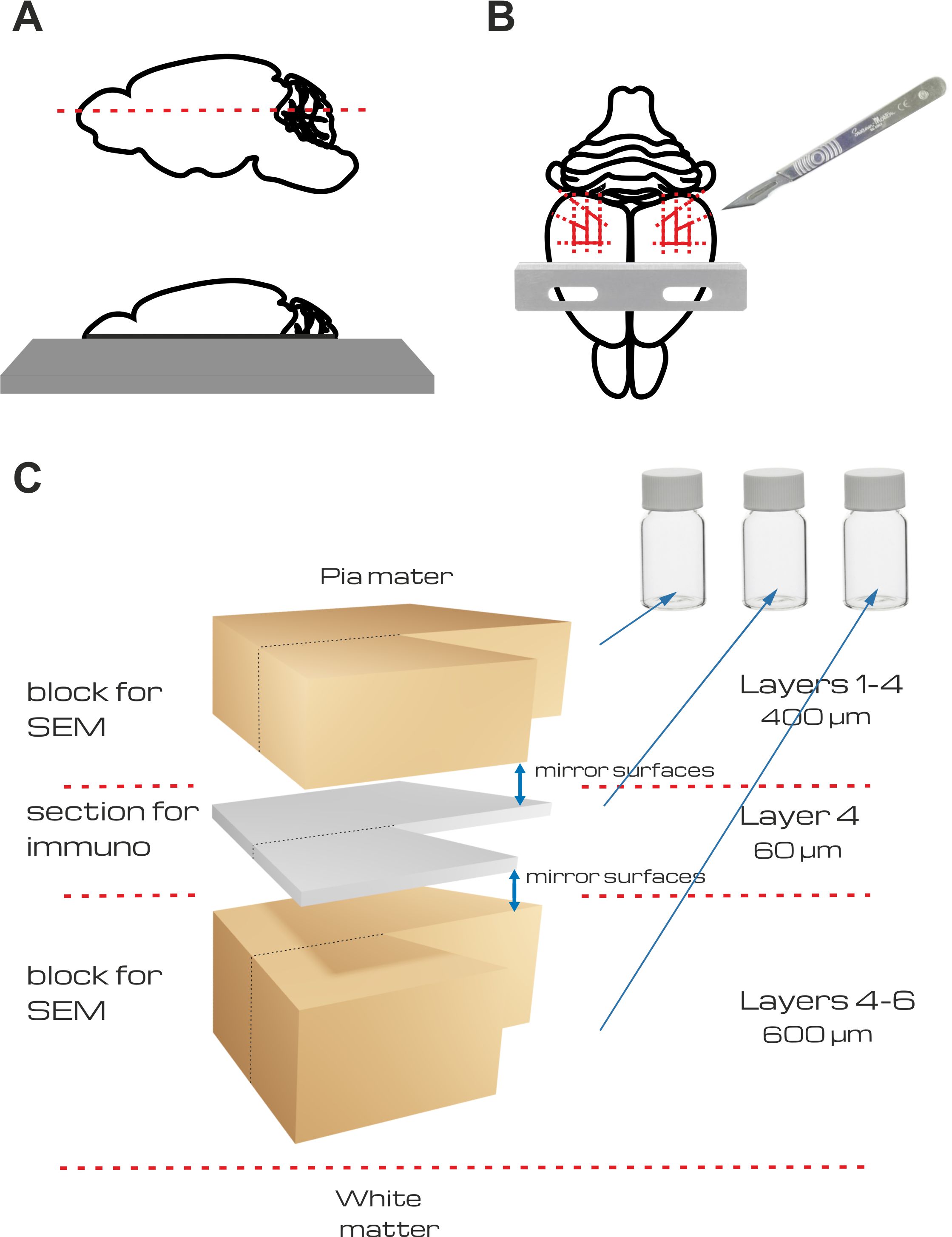

Supplement: Supplementary file 1 — Supplementary file1 (JPG 304 kb) [file 429_2022_2506_MOESM1_ESM.jpg]

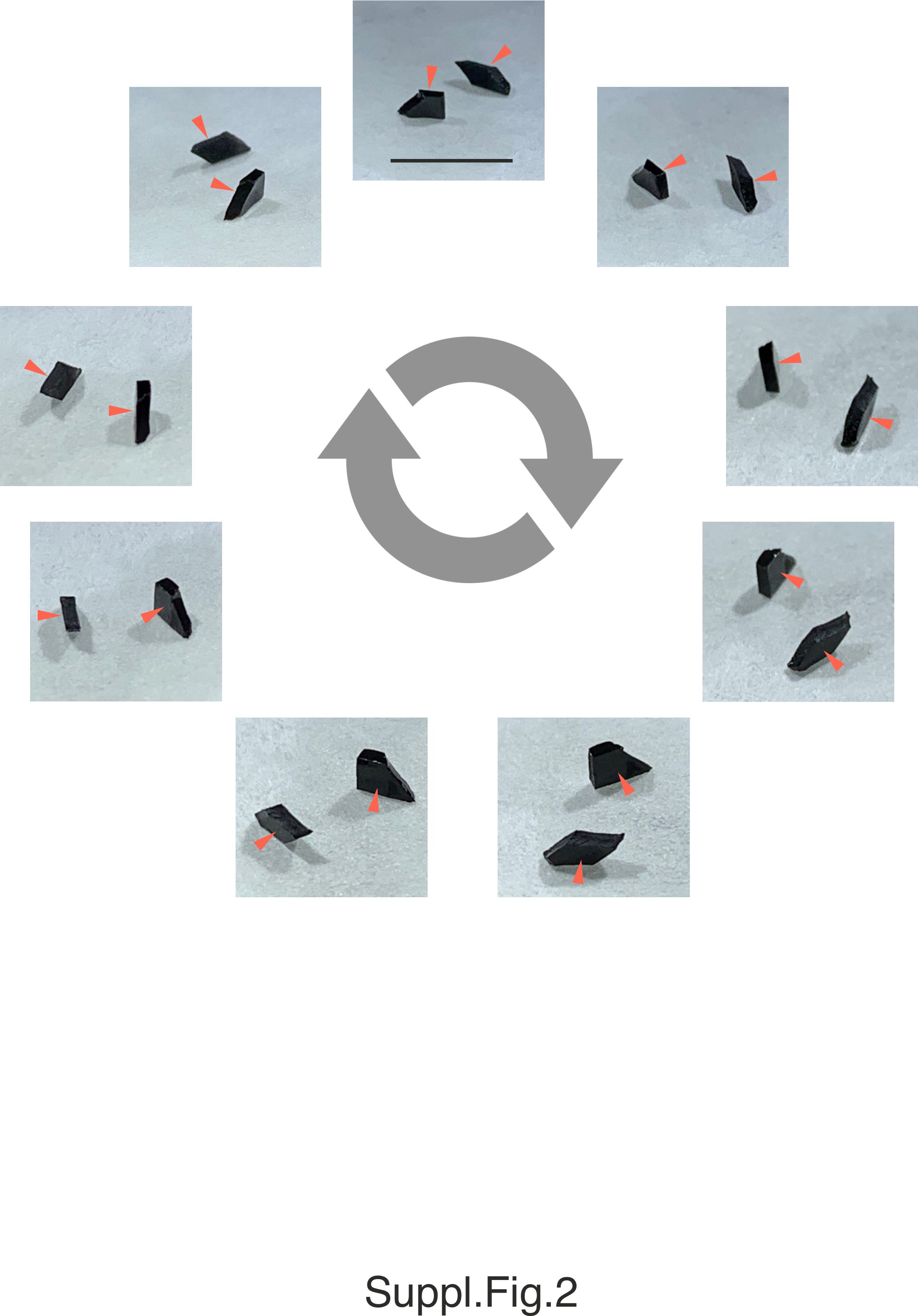

Supplement: Supplementary file 2 — Supplementary file2 (JPG 284 kb) [file 429_2022_2506_MOESM2_ESM.jpg]
